# Supplementary material for: Clinical application of tumour-in-normal contamination assessment from whole genome sequencing
Source: Nat Commun. 2024 Jan 18;15:323. doi: 10.1038/s41467-023-44158-2 (PMC10796348; doi:10.1038/s41467-023-44158-2)
Supplement: Supplementary file 3 — Reporting Summary [file 41467_2023_44158_MOESM3_ESM.pdf]

nature portfolio

Corresponding author(s): Alona Sosinsky and Giulio Caravagna

Last updated by author(s): Oct 10, 2023

Reporting Summary

Nature Portfolio wishes to improve the reproducibility of the work that we publish. This form provides structure for consistency and transparency in reporting. For further information on Nature Portfolio policies, see our Editorial Policies and the Editorial Policy Checklist.

Statistics

For all statistical analyses, confirm that the following items are present in the figure legend, table legend, main text, or Methods section.

n/a	Confirmed	
☐	☒	The exact sample size (*n*) for each experimental group/condition, given as a discrete number and unit of measurement
☐	☒	A statement on whether measurements were taken from distinct samples or whether the same sample was measured repeatedly
☐	☒	The statistical test(s) used AND whether they are one- or two-sided<br>*Only common tests should be described solely by name; describe more complex techniques in the Methods section.*
☐	☒	A description of all covariates tested
☐	☒	A description of any assumptions or corrections, such as tests of normality and adjustment for multiple comparisons
☐	☒	A full description of the statistical parameters including central tendency (e.g. means) or other basic estimates (e.g. regression coefficient) AND variation (e.g. standard deviation) or associated estimates of uncertainty (e.g. confidence intervals)
☐	☒	For null hypothesis testing, the test statistic (e.g. *F*, *t*, *r*) with confidence intervals, effect sizes, degrees of freedom and *P* value noted<br>*Give P values as exact values whenever suitable.*
☐	☒	For Bayesian analysis, information on the choice of priors and Markov chain Monte Carlo settings
☐	☒	For hierarchical and complex designs, identification of the appropriate level for tests and full reporting of outcomes
☐	☒	Estimates of effect sizes (e.g. Cohen's *d*, Pearson's *r*), indicating how they were calculated

Our web collection on statistics for biologists contains articles on many of the points above.

Software and code

Policy information about availability of computer code

Data collection	Available at Genomics England, as specified in the paper where instructions for data access are provided.
Data analysis	The software is available at https://github.com/caravagnalab/TINC (open source code) and at https://caravagnalab.github.io/TINC/ (website). A release at Zenodo is also linked withe paper (https://zenodo.org/records/10021433), together with a Source Data file.

For manuscripts utilizing custom algorithms or software that are central to the research but not yet described in published literature, software must be made available to editors and reviewers. We strongly encourage code deposition in a community repository (e.g. GitHub). See the Nature Portfolio guidelines for submitting code & software for further information.

Data

Policy information about availability of data

All manuscripts must include a data availability statement. This statement should provide the following information, where applicable:

- Accession codes, unique identifiers, or web links for publicly available datasets
- A description of any restrictions on data availability
- For clinical datasets or third party data, please ensure that the statement adheres to our policy

Samples are made available through the Genomics England datasets, and were analysed within the Genomics England Research Environment secure data portal, under Research Registry project code RR-360. The Genomics England data set can be accessed by joining the community of academic and clinical scientists via the Genomics England Clinical Interpretation Partnership (GeCIP), https://www.genomicsengland.co.uk/about-gecip/. To join a GeCIP domain, we reported the main

steps in the manuscript.

Research involving human participants, their data, or biological material

Policy information about studies with human participants or human data. See also policy information about sex, gender (identity/presentation), and sexual orientation and race, ethnicity and racism.

Reporting on sex and gender	Does not apply.
Reporting on race, ethnicity, or other socially relevant groupings	Does not apply.
Population characteristics	Does not apply.
Recruitment	The participants were recruited across 13 NHS Genomic Medicine Centres and written informed consent was obtained from the participants.
Ethics oversight	Approval for the 100,000 Genomes Genomics England project was obtained from the national research ethics committee (IRAS ID 166046). Participants were selected on the basis of having been identified by health care professionals and researchers within the NHS as having a cancer diagnosis.

Note that full information on the approval of the study protocol must also be provided in the manuscript.

Field-specific reporting

Please select the one below that is the best fit for your research. If you are not sure, read the appropriate sections before making your selection.

☒ Life sciences ☐ Behavioural & social sciences ☐ Ecological, evolutionary & environmental sciences

For a reference copy of the document with all sections, see nature.com/documents/nr-reporting-summary-flat.pdf

Life sciences study design

All studies must disclose on these points even when the disclosure is negative.

Sample size	Samples available from the 100.000 Genome Project at Genomics England.
Data exclusions	Nothing specific to this study.
Replication	Validations of computational predictions were made, where possible, via MRD testing.
Randomization	Does not apply.
Blinding	Does not apply.

Reporting for specific materials, systems and methods

We require information from authors about some types of materials, experimental systems and methods used in many studies. Here, indicate whether each material, system or method listed is relevant to your study. If you are not sure if a list item applies to your research, read the appropriate section before selecting a response.

Materials & experimental systems

n/a	Involved in the study	
☒	☐	Antibodies
☒	☐	Eukaryotic cell lines
☒	☐	Palaeontology and archaeology
☒	☐	Animals and other organisms
☒	☐	Clinical data
☒	☐	Dual use research of concern
☒	☐	Plants

Methods

n/a	Involved in the study	
☒	☐	ChIP-seq
☐	☒	Flow cytometry
☒	☐	MRI-based neuroimaging

Flow Cytometry

Plots

Confirm that:

☒ The axis labels state the marker and fluorochrome used (e.g. CD4-FITC).

☒ The axis scales are clearly visible. Include numbers along axes only for bottom left plot of group (a 'group' is an analysis of identical markers).

☒ All plots are contour plots with outliers or pseudocolor plots.

☒ A numerical value for number of cells or percentage (with statistics) is provided.

Methodology

Sample preparation

A sequential gating strategy is applied at diagnostic bone marrow sample to establish and define patients Leukaemia-associated aberrant phenotype (LAIP) using comprehensive 8 colour panels (8-9 antibodies for B-ALL)

Instrument

BD FACS Canto II instrument

Software

FACS DIVA software (BD)

Cell population abundance

MRD events found in the final LAIP gate are reported as a percentage divided by the total number of CD45 positive "live" WBC events analysed. A "different from normal" approach is also utilised when LAIP is similar to normal BM haematopoiesis or where there is marked regeneration in LAIP gates at later time points.

Gating strategy

B-ALL MRD
CD19, CD20, CD66c/CD123, CD38, CD10, CD45, CD34, CD81
CD19, CD20, CD73/CD304, CD38, CD10, CD45, CD34, CD81

AML MRD
CD45, CD34, CD13, CD33, HLA DR, CD117, CD11b, CD15
CD45, CD34, CD56, CD33, CD38, CD117, CD11b, CD15

T-ALL MRD
CD56/TCR alpha/beta/TCR gamma/delta (cocktail), CD99, CD45, CD5, CD7,CD3, CD4, CD8
CD56/TCR alpha/beta/TCR gamma/delta (cocktail), CD2, CD45, CD5, CD7,CD3, CD4, CD8

☒ Tick this box to confirm that a figure exemplifying the gating strategy is provided in the Supplementary Information.
